# The impact of protein-conjugate polysaccharide vaccines: an endgame for meningitis?

**DOI:** 10.1098/rstb.2012.0147

**Published:** 2013-08-05

**Authors:** Martin C. J. Maiden

**Affiliations:** Department of Zoology, University of Oxford, South Parks Road, Oxford OX1 3PS, UK

**Keywords:** *Neisseria meningitidis*, *Haemophilus influenzae*, *Streptococcus pneumoniae*, herd immunity, vaccines, population biology

## Abstract

The development and implementation of conjugate polysaccharide vaccines against invasive bacterial diseases, specifically those caused by the encapsulated bacteria *Neisseria meningitidis*, *Haemophilus influenzae* and *Streptococcus pneumoniae*, has been one of the most effective public health innovations of the last 25 years. These vaccines have resulted in significant reductions in childhood morbidity and mortality worldwide, with their effectiveness due in large part to their ability to induce long-lasting immunity in a range of age groups. At the population level this immunity reduces carriage and interrupts transmission resulting in herd immunity; however, these beneficial effects can be counterbalanced by the selection pressures that immunity against carriage can impose, potentially promoting the emergence and spread of virulent vaccine escape variants. Studies following the implementation of meningococcal serogroup C vaccines improved our understanding of these effects in relation to the biology of accidental pathogens such as the meningococcus. This understanding has enabled the refinement of the implementation of conjugate polysaccharide vaccines against meningitis-associated bacteria, and will be crucial in maintaining and improving vaccine control of these infections. To date there is little evidence for the spread of virulent vaccine escape variants of the meningococcus and *H. influenzae*, although this has been reported in pneumococci.

## 1. Introduction

Throughout the nineteenth and twentieth centuries ‘meningitis’ was one of the most feared infectious diseases, and it remains high both in public perception and as a public heath priority [1]. Clinically meningitis is an inflammation of the meninges, the membranes that surround the brain, which can have many causes including infection with bacteria, fungi, protozoa and viruses [2]. In popular understanding, however, ‘meningitis’ means one of a number of invasive bacterial diseases, especially those caused by the encapsulated bacteria *Haemophilus influenzae, Neisseria meningitidis* (the meningococcus) and *Streptococcus pneumoniae* (the pneumococcus, which also causes other diseases not further discussed here) [1]. These bacteria can cause severe, rapidly-progressing disease syndromes, usually in children and young adults, which may involve meningitis. The fearsome reputation of ‘meningitis’ in this sense is because, in the absence of rapid and effective treatment, it is often fatal and disabling sequelae are common in those who survive [3].

Antimicrobial agents are very effective against these diseases [3] and vaccines were available for much of the second half of the twentieth century, but it was the development of conjugate protein–polysaccharide vaccines which has brought the prospect of effective disease control and the potential for an ‘endgame’ [4]. The first such vaccines to be used were the *H. influenzae* serotype b (Hib) vaccines [5,6], followed by vaccines against serogroup C meningococci (MenC) [7], multivalent vaccines against pneumococci [8–10] and vaccines against other meningococcal serogroups including the MenAfriVac serogroup A vaccine [11]. These vaccines have led to marked reductions in invasive disease, have excellent safety and efficacy profiles, and are extremely effective in settings where high rates of immunization are attained [4,12].

Although conceived with the goal of individual protection it is now appreciated that the impact of these vaccines on disease is largely due to their effectiveness against asymptomatic carriage of these bacteria [4]. This reduces transmission, resulting in herd immunity, the protection of the unvaccinated [1]; however, the effect on carriage can, potentially, result in the evolution of virulent vaccine escape variants [13]. This article will examine these phenomena from a biological perspective, using the conjugate meningococcal vaccines as examples. The broader implications of our improved understanding will be discussed, illustrating how knowledge of the biology and natural history of these bacteria is central to the effective use of these excellent vaccines.

## 2. The biology of the encapsulated bacteria

The three ‘meningitis’ bacteria are genetically unrelated to each other yet they share a number of features other than causing similar disease syndromes [4]. All three are normally harmless members of the microbiota of the human naso- and oropharynx [14], which are spread person-to-person and have no known animal reservoir [15]. All can be encapsulated with polysaccharide coats that exist in a variety of antigenically distinct forms, some of which mimic human polysaccharides and are poorly recognized by the immune system [1,12]. The capsules, which are likely to have evolved to promote transmission among hosts, perhaps by preventing desiccation and/or protecting the bacteria from damage by UV light during aerosol transmission [16], are also key elements in disease causation, as only those bacteria that express a capsule are likely to cause disease [17]. The primary reason for this is that some, but not all, of these capsules are anti-opsonizing, allowing the bacteria to evade immune killing [18]. Once they have invaded host tissues, these organisms can spread systemically and rapidly as a bacteraemia and infect secondary sites such as the meninges and cerebrosprinal fluid. The capsules are, therefore, virulence determinants, i.e. they enhance virulence of the bacteria that express them; however, while necessary for disease causation, capsule expression is not sufficient for pathology. The majority of infections even with encapsulated variants result in asymptomatic colonization with no overt pathology [19,20].

In most human populations asymptomatic age-dependent carriage of these bacteria is common while invasive disease is rare. Disease is inimical to their onward transmission as it removes hosts from the population, effectively reducing the period of infectiousness. This presents a paradox—if it is no advantage to them, why do these bacteria cause disease? This is not fully resolved, but can be explained by the concept of the ‘accidental pathogen’: the evolution of colonization factors that promote disease may provide sufficient benefit during most colonization events that the reduction in fitness imposed by the occasional case of invasive disease is outweighed, and the virulence-promoting characteristic can spread in the population [19,20].

Populations of meningococci, pneumococci and *H. influenzae* are genetically and antigenically diverse. This at least partly due to the fact that they are naturally competent for transformation, meaning that they can take up DNA from their environment and incorporate it into their chromosomes [21]. This property is important in shaping the antigenic variation of capsules. These complex carbohydrates require multiple enzymes for their synthesis, which are encoded in single genomic regions, with each cell able to produce one type of capsule [22–24]. Competence provides a mechanism whereby genes in the capsular region can be replaced, resulting in an organism with the same genotype as the parent cell, but expressing a different capsule [25].

High-frequency lateral gene transfer affects more than the capsule locus and has a major impact on the population biology and evolution of these bacteria [26]. As bacteria reproduce asexually, it was long thought that they possessed a clonal population structure, with most inheritance occurring by descent, or vertically [27]. It is now appreciated that many bacteria indulge in ‘localized sex’ [28]. In this process, fragments of the chromosome are mobilized among bacteria that do not necessarily share a common ancestor, resulting in population structures that cannot be modelled by tree-like phylogenies [29]. Further, genes and consortia of genes such the capsule-encoding regions can spread through populations rapidly [30] so that linkage among phenotypic characteristics encoded by different parts of the genome is not necessarily imposed by descent.

Multilocus sequence typing [31] was developed to investigate the population structure of recombining bacteria. Using the same principle as multilocus enzyme electrophoresis [32], it indexes variation at multiple housekeeping loci, i.e. genes under stabilizing selection for conservation of metabolic function. The assignment of unique, but arbitrary, numbers to allelic variants accounts for the fact that genes may vary by mutational or by recombination events. The allelic variants at all loci examined, typically seven, are combined to form sequence type (ST). For many bacteria, including the meningitis organisms, these STs cluster in groups or ‘clonal complexes’ which are associated with phenotypic properties of clinical importance such as propensity to cause disease, expression of vaccine antigens or antimicrobial resistance. Most meningococcal disease is caused by a limited number of these clonal complexes, the ‘hyperinvasive lineages’ [33–35].

## 3. The development of meningococcal polysaccharide vaccines

The modern era of meningococcal vaccines began in the late 1960s, triggered by the evolution of resistance to available chemotherapies [36]. Meningococcal disease was first described at the beginning of the nineteenth century [37], with outbreaks reported globally over the succeeding 100 years [38]. The disease has been frequently reported in the military, especially in recruit camps, presumably because of increased transmission in the cramped conditions prevailing and the presence of rural recruits with low immunity to at least some meningococci [39]. Outbreaks in the British Army in the First World War led to some of the earliest studies of meningococcal carriage and transmission and their relationship to disease outbreaks [40]. It is now well established that most cases of invasive meningococcal disease occur shortly after an individual has acquired a novel meningococcus [41], presumably as a consequence of a dysfunctional or failed attempt by the bacterium to establish colonization [42].

Attempts to interrupt transmission by changing factors such as the distance between beds in barracks [40] met with inconsistent success and during the Second World War the problem of meningococcal disease among recruits and in the populations of the combatant countries were severe [39,43]. In the military these problems were addressed by the prophylactic use of sulfonamide drugs, which had been developed in the 1930s, replacing serum therapy for meningococcal disease treatment [44]. These antimicrobials were effective in eliminating meningococci from asymptomatic carriage, resulting in reduced transmission. In a setting such as a military recruit camp, where the at-risk group is easily defined in space and time, and compliant with treatment, this is a highly effective intervention and, in the post-war period, mass sulfadiazine prophylaxis successfully prevented meningococcal disease among recruits in the US Army [45].

**Figure 1.**
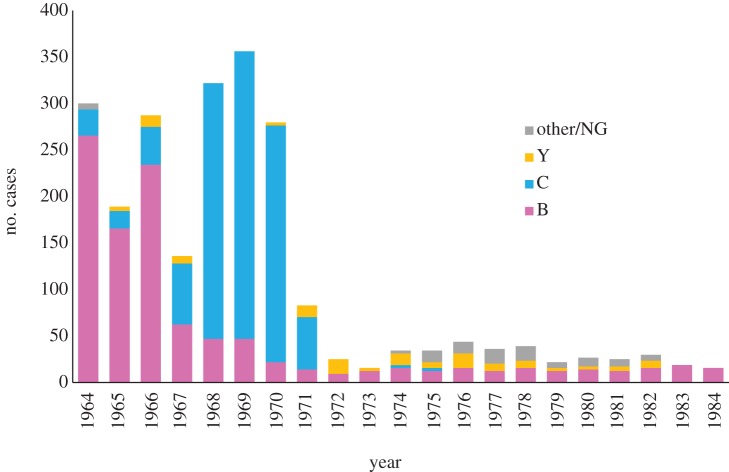
Evolution of meningococcal disease in the US Army in the Vietnam War era. The disease outbreak was predominantly caused by sulphonamide-resistant organisms belonging to the ST-11 (ET-37 complex), with a capsule replacement event that replaced sulfonamide-resistant serogroup B meningococci with sulfonamide-resistant serogroup C organisms. Plain polysaccharide vaccines against serogroup C were introduced in 1971, against serogroup C and A in 1978, and against serogroups A, C Y and W in 1982. Data compiled from Brundage & Zollinger [45] and Wang *et al.* [50]. NG, not-groupable isolates.

There were two drawbacks to this intervention: first, while effective in closed and semi-closed communities, when the at-risk period is easily identified, widespread use of chemical prophylaxis is not an intervention that can be used routinely on a population scale. Prophylaxis in the wider community can be used when localized outbreaks have been identified, but defining the extent of the group to be treated can be difficult [46]. This intervention cannot be used in cases of hyperendemic meningococcal disease, where an outbreak remains in a community for many months or years, or when the outbreak is geographically dispersed. Further, where they clear carriage, widespread administration of antimicrobials can lead to the emergence of resistance [47]. Not all antimicrobials affect carriage and the use of these agents does not lead to the emergence of resistant strains; for example, although penicillins are effective in disease treatment they do not affect carriage [46], and reduced susceptibility to these antimicrobials is yet to become a therapeutic problem [48]. Resistance to sulfonamides arises by a number of mechanisms and is now widespread in meningococci [49], and they have been replaced for treatment of both meningococcal carriage and disease. During the Vietnam War, large outbreaks of sulfonamide-resistant meningococcal disease occurred among recruits in the US Army and Navy training camps in California, first caused by serogroup B organisms and subsequently by closely related serogroup C organisms [45] (figure 1).

An influential set of investigations into the biology of meningococcal disease were undertaken in response to this emergency, which have become paradigms for the development of meningococcal vaccines in particular and encapsulated bacteria generally [51–55]. *In vitro* assays demonstrated that most human adults have circulating antibodies capable of killing meningococci (‘bactericidal antibodies’) [51]. The distribution of disease in the human population was inversely correlated with this bactericidal activity, with the age-group at most risk of disease, young children aged six months to 1 year, exhibiting the lowest levels, as a consequence of the waning of maternally acquired immunity before the development of adaptive immunity [52]. Tests with meningococci belonging to different serogroups showed that these antibodies were specific to particular capsules, and that the level of bactericidal effect corresponded to the degree of protection against disease [53].

These studies led to the use of purified bacterial polysaccharide vaccines: the ‘plain’ polysaccharide vaccines [53], first against serogroup C meningococci [55] and subsequently against serogroups A, W and Y. These vaccines had an excellent safely profile and were effective in the military setting, their introduction preventing meningococcal disease caused by serogroup A, C, Y and W bacteria (figure 1) [39]. Unfortunately, it was not possible to extend this success to serogroup B meningococci [56] as this polysaccharide is poorly immunogenic, probably because of its structural similarity to host polysaccharides that decorate the neural cell adhesion molecule of human foetal tissues [57]. A further concern with this polysaccharide is that effective vaccines may lead to autoimmune reactions [57], and the inclusion of this antigen in vaccine preparations remains controversial and unlikely in the foreseeable future [58].

The plain polysaccharide vaccines did not resolve the problem of meningococcal disease in the community. Bacterial polysaccharide capsules have evolved, at least in part, to evade the mammalian immune responses, and their repeating sugar structures are poorly recognized by the human immune system. Immune responses against these antigens do not invoke T-cell help and do not result in affinity maturation or the generation of immunological memory [1]. Consequently, plain polysaccharide vaccines elicit only primary immune responses comprising low-affinity IgM antibody and subsequent immunization does not generate a secondary response; indeed, repeated immunization can result in hyporesponsiveness, as primary B cells with affinity to the polysaccharide are exhausted [59,60]. The lack of T-cell involvement in the immune response therefore has a number of important consequences: (i) the vaccine works poorly or not at all in young children, a major at-risk group, and (ii) no memory response is generated in adults [61]. Plain polysaccharide vaccines are not suitable for use in infant immunization programmes for this reason [62,63] and even in adults they have to be repeatedly administered. They are also ineffective against carriage, having at best a short-term effect [55,64–67], so, while effective in the short-term in a closed community setting, these vaccines are not suitable for population-scale interventions outside of epidemics or for infant immunization.

## 4. The development and introduction of meningococcal serogroup C conjugate vaccines

The invention of conjugate vaccines in the 1980s was a major breakthrough in polysaccharide vaccine development: these vaccines contain a polysaccharide molecule, chemically conjugated to a T-cell-stimulating antigen, such as the diphtheria or tetanus toxoids [68]. This has the effect of recruiting T-cell help and, therefore, results in the generation of affinity-matured immunological responses and immunological memory [1]. As with the plain polysaccharide vaccines, these preparations have an excellent safely profile but have the advantage of being immunogenic in small children as well as adults, making them suitable for population-scale interventions. They are more expensive to produce as they contain at least two molecules which have to be prepared and chemically linked, or conjugated [69].

**Figure 2.**
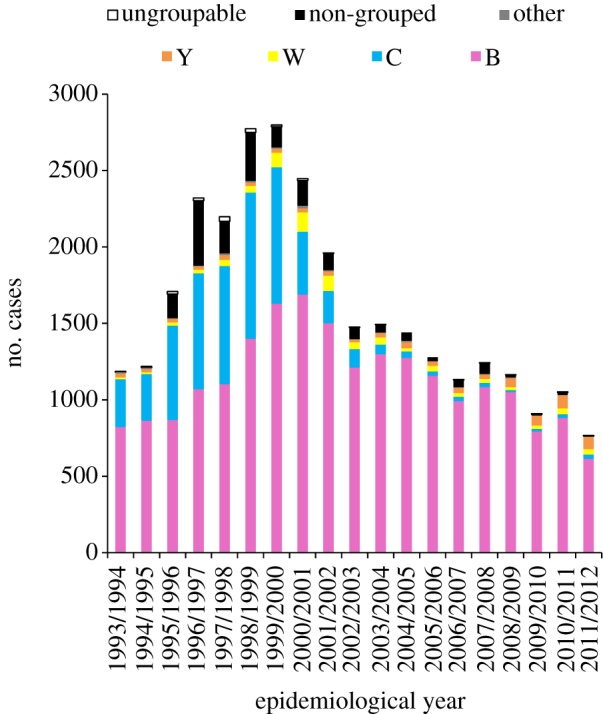
The number of laboratory confirmed cases of meningococal disease in England and Wales before and after the introduction of MCC vaccines in autumn 1999. Drawn with data from Gray *et al.* [71] and the UK Health Protection Agency (http://www.hpa.org.uk/Topics/InfectiousDiseases/InfectionsAZ/MeningococcalDisease/EpidemiologicalData/). Ungroupable isolates were those samples for which no serogroup could be obtained (e.g. as a result of non-culture diagnosis without serogroup determination).

Following the successful introduction of the *H. influenzae* serotype b conjugate (Hib) vaccines in the early 1990s [70], meningococcal serogroup C conjugate (MCC) polysaccharide vaccines became available in the late 1990s, at a time of elevated levels of serogroup C meningococcal disease. This epidemic was caused by the global spread of serogroup C ST-11 ‘ET-15' complex meningococci (figure 2), and was all the more alarming as it was characterized by (i) elevated levels of disease in older adolescents and young adults, and (ii) localized outbreaks in educational settings such as residential universities [72]. In response to this problem, the UK Department of Health implemented an accelerated introduction of the MCC vaccines. A single-valent MCC vaccine introduction was undertaken as virtually all meningococcal disease in the UK in the late 1990s was caused by serogroup B or C organisms and no serogroup B vaccine was available (figure 2) [73].

The MCC vaccines were licensed on the basis of serological correlates of protection; phase III efficacy trials were not considered owing to the sporadic nature of the disease and epidemiological situation at the time. The likely effectiveness of the vaccine was, therefore, assessed in clinical trials which measured immunological responses, particularly the generation of bactericidal antibodies [74]. These studies suggested that bactericidal immune responses were elicited in all age groups. Given the age profile of those infected, principally infants, adolescents and young adults, the vaccine was introduced into the routine infant immunization schedule with a school-based ‘catch-up’ campaign covering individuals up to 18 years of age; this was later extended to older young adults but, as this could not be administered through schools, the coverage achieved for these age groups was lower [75]. An enhanced disease surveillance programme was put in place to monitor the effects of vaccine introduction [73].

As with the earlier Hib vaccine, the UK MCC vaccination introduction was dramatically successful, with rapid reductions in meningococcal serogroup C disease in vaccinated and unvaccinated individuals [76] that have been sustained for more than a decade [77] (figure 2). This success stimulated the introduction of MCC vaccines in a number of other countries and provides the prospect of serogroup C meningococcal disease control [78–80]. A number of studies conducted since the introduction of the vaccines have resulted in changes to the UK vaccination schedule, principally in light of the realization that carriage effects were more important than induction of immunological memory in individuals [81]. This raises the interesting question as to whether effects on carriage are suitable, and indeed preferable, endpoints for vaccine efficacy trials.

## 5. Population effects: herd immunity and vaccine escape

The effect of MCC vaccines on carriage was not known at the time of introduction [13], although previous experience with the Hib conjugate vaccines suggested that the immunization with MCC vaccines would reduce carriage of group C meningococci [82]. For accidental pathogens, such as *H. influenzae* and the meningococcus, a vaccine that prevents disease, but has no impact on carriage, could have a very high level of efficacy, while being less useful per vaccinated person owing to the lack of herd immunity. On the other hand, preventing carriage, while essential to herd immunity, potentially selects for both (i) capsule variants derived from strains expressing the targeted serogroups, as well as (ii) strains that compete with them but which are not targeted by the vaccine [83]. The negative consequence of this, vaccine escape, is the evolution or spread of variants that are not affected by the vaccine-induced immunity, and are released from competition with those variants that are affected. Both of these phenomena can be induced by vaccination campaigns.

The level of population immunity generated by immunization will be a product of the efficacy of the vaccine against transmission and the vaccine coverage achieved. For infectious agents with no antigenic variation such as measles, or famously smallpox [84], high-levels of population immunity can lead to the removal of the infectious agent and disease eradication or extinction. However, where organisms have the capacity to vary their antigens, either by mutation, phase variation or lateral gene transfer, the selection pressures imposed by vaccine-induced immunity can lead to the emergence and/or spread of novel variants that escape vaccine control [13]. Vaccine efficacy against carriage is therefore a two-edged sword, and whether it ultimately acts beneficially or harmfully depends on the biology of the agent being protected against and the mechanisms of immunity. Prior to the introduction of MCC vaccines, it was known that hyperinvasive meningocoocci could alter their capsules by lateral gene transfer of a single gene in the capsule locus, and that this had happened during the US Army outbreaks. This also involved ST-11 complex meningococci, although the change had been from serogroup B to serogroup C, a direction that had actually promoted the impact of vaccines on the epidemic (figure 1) [45].

## 6. The UK Meningococcal Carriage study

To assess the population effects of the MCC vaccines, data on the prevalence of serogroup C ST-11 complex meningococci among asymptomatic carriers were required, in addition to information on the meningococci causing disease, which was being collected by enhanced disease surveillance. The UK Meningococcal Carriage (UKMenCar) study was initiated [85] to collect sufficient carriage data by measuring the point prevalence of meningococci among children and young adults aged 15–19 attending full-time education. This was done immediately prior to, and for 2 years after, the introduction of MCC vaccines. The cohort was chosen as it was the first to receive the vaccine, was accessible as the vaccines were being administered in schools and colleges, and was known to carry meningococci at high rates. The latter was particularly important as, although meningoccal carriage is relatively common, averaging about 10 per cent of the population across all age groups, carriage of the epidemic serogroup C ST-11 clonal complex strain was very rare. Powered to detect changes in the carriage of the epidemic bacteria, the UKMenCar study aimed to isolate carried meningococci from sufficient individuals in each year (estimated to be 16 700) and to characterize them (i) phenotypically, for the expression of capsular antigens, and (ii) genetically, to identify their capsule-encoding genes and clonal complex. Simultaneously, information was gathered on the risk factors for meningococcal carriage including demographic and behavioural data. In the first year of the study individuals were sampled immediately after the vaccine was administered, and in subsequent years the same cohorts (school and college year groups) were surveyed around the anniversary of vaccine introduction [86].

**Figure 3.**
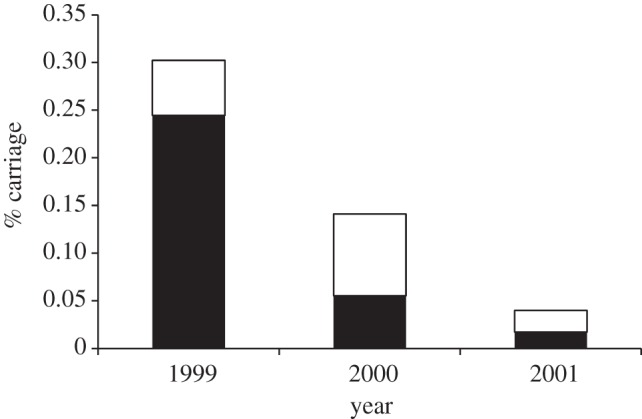
The fate of the epidemic meningococcal strain serogroup C, ST-11 complex, in carriage by UK teenagers (aged 15–19 years) before (1999) and for two years subsequent to the introduction of MCC vaccines in the UK [86,87]. Black represents ST-11 complex meningococci that were expressing their capsule and white indicates ST-11 complex isolates that contained the serogroup C capsular region but did not express it. Note the very low rates of carriage of the epidemic strain even before vaccine introduction.

The study showed that, while the prevalence in carriage of the epidemic serogroup C ST-11 complex meningococci was indeed low, the MCC vaccine reduced this by around 80 per cent over the 2 years [85–87] (figure 3). The continued decline over successive years was consistent with a herd immunity effect as the vaccinated cohorts aged year-by-year, increasing vaccine coverage among young adults. The study demonstrated that meningococci belonging to the epidemic clone were especially affected, probably because they expressed their capsules at high rates [86]. Many meningococci down-regulate capsule expression during asymptomatic carriage, with rates of expression of capsules of isolates recovered from the nasopharynx ranging from 10 to 100 per cent; however, more than 60 per cent of the serogroup C ST-11 complex meningococci recovered in the UK carriage study showed capsule expression, with an even higher proportion (81%) expressing their capsules before vaccine introduction, which was consistent with their carriage being particularly affected by MCC vaccine-induced immunity [87] (figure 3).

The study further demonstrated that the rapid increase in carriage of meningococci seen in late teenage and early adulthood [88–90] is due to age-related changes in social behaviour: regular attendance at pubs and clubs, smoking and number of kissing partners were all positively correlated with increased carriage rates [91]. These observations were consistent with the higher carriage and increased meningococcal disease consistently observed among individuals in regular close contact with others, e.g. those living in closed and semi-closed communities. The UK MCC immunization programme, which achieved very high coverage rates of individuals aged 18 and under, ensured that by autumn 2001 most people in the UK under the age of 20 had received a MCC vaccine. This amplified the benefits of the vaccine by reducing the circulation of serogroup C meningococci among the group in which most transmission occurred, although the organism was not eliminated even in this group [81]. A similar effect was achieved by the Dutch implementation of MCC vaccines, which administered a single dose to all individuals aged between 14 months and 18 years [92].

The high impact of the MCC vaccines on the carriage of serogroup C ST-11 meningococci raised the possibility of the emergence of virulent vaccine escape variants [13], which could be mediated by a number of possible mechanisms including (i) the spread of virulent ST-11 meningococci with different capsules, and (ii) the replacement of the ST-11 meniningococci by a virulent strain with a distinct serogroup–genotype combination. In both cases these variants would have to be in competition with the epidemic strain, either by direct competition for a particular niche in the nasopharynx or by antigenic cross-immunity mediated by antigens not included in the vaccine. Vaccine escape could, therefore, emerge either by the evolution of novel variants or the spread of pre-existing meningococci with these characteristics; however, ten years after MCC vaccine introduction, no such events have occurred. Virulent capsule variants of the ST-11 clonal complex exist, but they have not replaced the serogroup C ST-11 complex meningococci in epidemic disease. Even the introduction of serogroup W ST-11 complex meningococci into the UK, as a consequence of the Hajj outbreaks in early 2001, resulted in only a limited amount of disease [93]. The virulent nature of the serogroup W ST-11 complex meningococci is underlined by the fact that they caused about 5 per cent of all meningococcal disease in the UK in 2010/2011 [94] (figure 2), although these numbers are small compared with the height of the serogroup C ST-11 complex epidemic.

There are a number of explanations for the lack of replacement of the serogroup C ST-11 complex strain. It is possible that the factors that made the epidemic clone hyperinvasive, particularly the expression of serogroup C polysaccharide capsule, were also responsible for the successful transmission of the organism and that variants with different capsules were ineffective in exploiting the niche vacated by the epidemic strain. Another possibility is that the spread of the serogroup C ST-11 complex clone within the UK generated natural population immunity to its repertoire of subcapsular antigen variants, preventing the emergence of variants expressing a different capsule. However, it is also the case that our understanding of the niches exploited by these different variants during carriage and their relationships to invasive disease is rudimentary. In this respect it is pertinent to note that other serogroup C variants of the ST-11 clonal complex were circulating at the time of the vaccine introduction, as demonstrated by their presence in a University outbreak, although they did not contribute to the epidemic [95].

Apparent instances of capsule replacement with ST-11 complex meningococci were observed in some countries [96], but these strains did not become major public health problems. There was little evidence of the replacement of the serogroup C ST-11 complex meningococci with other hyperinvasive lineages in either disease or carriage [94]. An increase in serogroup B ST-213 complex meningococci, in both disease and carriage, was observed in the UK and a number of countries that introduced the vaccine, but this could have been due to natural fluctuations in the carriage of this genotype [87]. Up to the time of writing there is no evidence for a major negative impact of MCC vaccine introduction; however, in the longer term virulent ST-11 complex escape variants may arise, the serogroup B ST-11 complex meningococci that began the US Army outbreaks in the late 1960s providing a precedent [50] (figure 1).

In summary, the success of the MCC vaccines was a consequence of the interaction of the immune responses that they elicited with meningococcal transmission biology. The vaccines targeted the serogroup C polysaccharide antigen, which was both the major virulence determinant and expressed during carriage by the invasive genotype. This association meant that the vaccine effectively targeted the hyperinvasive epidemic meningococcal genotype (serogroup C ST-11 complex). Further, the immunization of individuals up to the age of 18 years of age resulted in the majority of individuals becoming immune to carriage of serogroup C meningococci for a prolonged period, resulting in a marked long-term reduction in the carriage prevalence of the epidemic strain and consequent reduction in disease levels (figures 2 and 3) [86,87].

## 7. Serogroup A vaccines for Africa

The successful introduction of the MCC vaccines stimulated interest in vaccination against the greatest burden of meningococcal disease internationally: the periodic, large epidemics occurring in the ‘meningitis belt’ of sub-Saharan Africa. These seasonal outbreaks, first reported in 1905 and systematically described by Lapeyssonnie in the mid-twentieth century [97], are typically caused by serogroup A meningococci and occur with a periodicity of 7–10 years. They frequently involve hundreds of thousands of cases and thousands of deaths. In addition to this large burden of morbidity and mortality, principally in children, the intensity of the outbreaks, which usually last a few weeks, increases their disruptive impact on health systems in low-income settings [98]. Attempts to control these outbreaks with plain polysaccharide vaccines were only partially successful as the vaccines had to be administered once an outbreak had been detected, requiring the maintenance and mobilization of large stockpiles of vaccines at short notice [98].

The Meningitis Vaccine Project (MVP), a partnership of the World Health Organization and the Programme for Appropriate Technology in Health, funded by the Bill and Melinda Gates Foundation, was formed to address this problem by the development of an affordable serogroup A conjugate vaccine [99]. This was achieved with the innovative formation of a ‘North–South’ partnership, with conjugate technology and vaccine components provided by North American and European partner's and vaccine production by the Serum Institute of India. The resultant product, a tetanus-toxin (TT) polysaccharide conjugate vaccine (TT-PSA, MenAfriVac), was produced, tested prequalified and introduced during the first decade of the twenty-first century [100].

As little information was available on the carriage of meningococci in Africa at the time of vaccine introduction [101], it was decided to immunize all individuals up to the age of 29 to ensure the maximum effectiveness [102]. The vaccine was first introduced in Burkina Faso in 2010, with enhanced disease surveillance and simultaneous carriage studies to monitor the impact of the vaccine [103]. As with the MCC vaccines, a rapid and dramatic effect was observed both on disease rates and on carriage of serogroup A meningococci although the carriage rates of these organisms were also very low even during epidemics [103–105]. At the time of writing, the continued rollout of MenAfriVac across the meningitis belt has presented the prospect of the elimination of epidemic serogroup A meningococcal disease [106]. For the maintenance of vaccine effectiveness, however, it is important know the rates and dynamics of carriage across the meningitis belt, which remains poorly understood and which is unlike meningococcal carriage in high-income countries [101,106]. The MenAfriCar consortium (http://www.menafricar.org) has worked to monitor the impact of vaccine introduction on the carriage of meningococci across the meningitis belt by means of pre- and post-vaccination carriage surveys and molecular characterization of the isolates obtained.

Although serogroup C and A meningococcal disease are distinct in their geographical distribution, seasonality, attack rate and scale, both are caused by certain clonal complexes that are associated with a particular capsule, the expression of which appears to be important for transmission, asymptomatic carriage and disease [107]. The continued success of vaccination against these organisms depends on the continued association of these characteristics. It is, unfortunately, not fully understood why these associations are so strongly maintained, even in the face of high levels of immunization and the potential for lateral gene transfer [108]. It is possible, and perhaps likely, that over time these particular meningococcal strains will be replaced in carrier populations with other hyperinvasive meningococci, perhaps associated with different serogroups, leading to renewed outbreaks of disease, and continued disease surveillance is required to assess this. In this respect, the lack of a comprehensive meningococcal vaccine remains a concern [109].

In summary, the introduction of the meningococcal conjugate polysaccharide vaccines is a continuing success in combating invasive meningococcal disease [73,102] as a consequence of their effectiveness in inducing herd immunity and not, at least up to the time of writing, leading to vaccine escape either by capsule switching (the acquisition of a novel capsule by the original pathogen strain) or replacement (the replacement of the original epidemic strain with a genetically and antigenically distinct strain) [13]. This lack of escape from vaccine control appears to be the consequence of a number of factors, including the induction of long-lasting immunity effective against asymptomatic carriage and the association of the invasive meningococci with expression of a particular capsular polysaccharide [86]. The extension of this paradigm to other meningococcal serogroups that are associated with disease (B, W, X, Y) would potentially result in the control and perhaps elimination of meningococcal disease [77,109]. This can likely be achieved for serogroups W, X and Y, and a number of such vaccines exist with others being developed, but there is little prospect of a polysaccharide vaccine against serogroup B meningococci, and it is not clear at the time of writing whether ‘group B substitute’ vaccines, which mostly contain protein antigens, would have a similar effect [67,109,110].

## 8. Experience with other conjugate polysaccharide vaccines

The Hib conjugate polysaccharide vaccines, which were introduced approximately a decade before the serogroup C meningococcal vaccines, exhibit similar properties [111]. In the case of *H. influenzae* type b, the age-range of those at risk of disease and the carrier population was lower. Consequently, the UK Hib vaccine introduction and catch-up campaign extended only up to those 48 months old, but this also resulted in a very dramatic decline in disease rates owing to the herd immunity elicited [112]. As with the meningococcal vaccines, some changes in vaccine schedule have been made to ensure the maintenance of herd immunity [113]. While there has been some increase in public heath interest in disease caused by non-typeable *H. influenzae*, and those expressing other capsular antigens [114], the serotype b organisms have not been replaced as major causes of meningitis and there has been little evidence for widespread vaccine escape [115].

While conjugate vaccines against the pneumococcus have been developed and successfully deployed [116], reducing levels of disease caused by the capsular serotypes present in the vaccine [4], their impact has been constrained by the number of capsular types of these organisms and the emergence of escape variants [117]. More than 90 capsular types have been described for the pneumococcus, about ten times the number seen in meningococci and *H. influenza*e, and many of these are associated with invasive disease. It has, therefore, been necessary to produce multivalent vaccines that now cover up to 13 different serogroups [10]. As the distribution of serotypes of pneumococci is different in different parts of the world [118], the implementation of these vaccines is complicated, as a particular combination may be required in one region but not be useful in another. More of a threat to these campaigns, however, is the fact that the association of serotype with genetic type is not as marked as has been seen with the serogroup C and A meningococci, and with *H. influenzae* type b. Consequently, there has been evidence of the spread of invasive pneumococcal genotypes with different serotypes, which can be linked to vaccine introduction followed by a particular invasive genotype becoming associated with a different capsular type [25]. It is not known at the time of writing why the biology of the pneumococcus is so different from that of the meningococcus and *H. influenzae* in this respect.

## 9. Conclusions and future prospects

The development and implementation of conjugate polysaccharide vaccines has reduced the burden of meningitis and related diseases in many countries and presents the prospect of a ‘meningitis free world’ [119]. Their success has come, not only from their excellent safety and immunogenicity profiles, but also as a consequence of their use in population-scale immunization campaigns that covered the cohorts which transmitted the disease, resulting in herd immunity. Where they have been introduced, the Hib and MCC vaccines have led to the control of serotype b *H. influenzae* and serogroup C meningocci, respectively, and there is at least the prospect of conjugate serogroup A polysaccharide vaccines having a similar impact for meningococcal disease in Africa. Disease caused by the vaccine serotypes of pneumococci has also been impacted, with the caveats surrounding the antigenic repertoire of these organisms and vaccine escape [117]. The total elimination of meningitis is perhaps unlikely, but with concerted vaccine use, control of the disease can be anticipated [109].

The degree to which these advances can be extended depends on the development and implementation of novel vaccines. For the meningococcus, the introduction of vaccines against serogroups W, Y and X is required. Some tetravalent (A, C, W, Y) vaccines are available [120] and there are moves to produce inexpensive versions of these for use in Africa with the MVP model. Serogroup B meningococci present more of a problem with little prospect of a conjugate polysaccharide vaccine against them owing to safety concerns [57]. With pneumococci, the large numbers of different capsular types, which are not uniformly distributed, make it not feasible to eliminate all serotypes everywhere, but the development of specific conjugate vaccines is one way that disease caused by this important pathogen could be further controlled [116].

As conjugate vaccines generate their most impressive results through population effects, epidemiological modelling has a continuing impact on the design and refinement of implementation programmes [121,122]. Modelling has been especially influential when factors such as the actual duration of protection are unknown; in these circumstances, a number of scenarios can be explored and the disease incidence followed to establish which scenario most closely resembles reality. With MCC vaccines this indicated that the protection against carriage after immunization was at the higher ends of expectation, with herd immunity maintained for at least 10 years [123]. This approach can also indicate when further intervention may be necessary [124] and is important in cost effectiveness studies, which have an increasing role in vaccines implementation [125].

In addition to an appropriate understanding of the duration of protection against carriage, and the age groups that need to be immunized, the maintenance of herd immunity also depends on the existence of an adequate healthcare infrastructure. Control of meningitis and related diseases has been most dramatic in those countries that have delivered rapid and very high coverage of vaccination of the age-groups in which transmission occurs, for example with the introduction of Hib vaccination in the UK, MCC in the UK and the Netherlands, and, probably, MenAfriVac in Burkina Faso. For these successes to be maintained, however, it is essential that adequate resource is available to maintain herd immunity in the transmitting cohort. It is important to note that this is not always the cohort most at risk of disease, which presents challenges both ethically and in terms of resource provision, as it may involve immunizing individuals against diseases which they are at very low or negligible risk of contracting.

In conclusion, conjugate polysaccharide vaccines against the encapsulated bacteria meet many of the requirements of ideal prophylactics as they (i) are safe and efficacious, (ii) target components of the bacteria that are required for virulence, (iii) reduce carriage, thereby limiting transmission and inducing herd immunity, and (iv) have long-lasting protective responses with immunological memory. Conjugate polysaccharide vaccines against serogroup C and A meningococci, *H. influenzae* type b, and certain serotypes of the pneumococcus have reduced disease to very low levels [125], although it remains unclear whether these pathogens can be eradicated by their use. Continued monitoring of vaccine escape variants of the pneumococcus will be required, as will monitoring against the possibility of such variants spreading in the meningococcus and *H. influenzae* populations. Therefore, despite the great successes of these vaccines over the past two decades, the endgame of the diseases that they have played an important role in controlling is likely to be protracted and demand continued research and surveillance.
